# Real-world treatment patterns, healthcare resource use and clinical outcomes of patients receiving second line therapy for advanced or metastatic gastric cancer

**DOI:** 10.1186/s12876-020-01232-z

**Published:** 2020-05-05

**Authors:** David Gómez-Ulloa, Mayur Amonkar, Smita Kothari, Winson Y. Cheung, Ian Chau, John R. Zalcberg, Núria Lara Suriñach, Alfredo Falcone

**Affiliations:** 1 Real World Solutions, IQVIA, Barcelona, Spain; 2 Merck & Co., Inc., North Wales, PA USA; 3 Merck & Co., Inc., Kenilworth, NJ USA; 4 British Columbia Cancer Agency, Vancouver, BC Canada; 5 Royal Marsden Hospital, London & Surrey, UK; 6 Alfred Health and School of Public Health, Monash University, Melbourne, Australia; 7 University of Pisa, Pisa, Italy

**Keywords:** Gastric cancer, Chemotherapy, Outcomes, Health care utilization

## Abstract

**Background:**

Second-line (2 L) chemotherapies for advanced or metastatic gastric cancer have shown improved survival but there is no commonly accepted standard of care. This study examines real-world patient characteristics, treatment patterns, healthcare resource use (HCRU) and clinical outcomes in this setting.

**Methods:**

Retrospective chart reviews were performed at participating institutions from Australia, Canada, Italy and UK for adult patients receiving 2 L treatment for advanced/metastatic disease from January 2013 to July 2015. Data were collected for 12 months or until death.

**Results:**

Two hundred eighty patients were included, mean age was 60.9 years and 68.9% were male. Half (51.8%) received monotherapy in 2 L, of whom 69.0% received taxanes. Irinotecan monotherapy was common in Australia (30.0% of monotherapy patients) and Canada (43.8%), but infrequent in Italy and UK. Doublet chemotherapy was used in 36.4% of 2 L patients, most commonly fluoropyrimidine + irinotecan. Use of targeted therapies (trastuzumab, ramucirumab) was infrequent except in Italy. Estimated median real-world progression-free survival (rwPFS) and real-world overall survival (rwOS) from the time of 2 L treatment initiation was 3.09 (95% CI: 2.76–3.68) and 6.54 (5.29–7.76) months, respectively, and estimated 12-month rwPFS and rwOS rate was 8 and 26%, respectively. Only a minority (26.8%) of patients were hospitalized during the follow-up period, with the lowest hospitalization in Italy (16.7%). Laboratory and imaging tests were performed for 93.2 and 70.4%, respectively.

**Conclusions:**

About half of patients received monotherapy as 2 L chemotherapy for advanced/metastatic gastric cancer and a third received doublets. Real-world clinical outcomes for 2 L treatment are poor and HCRU is considerable.

## Background

It is estimated that about 1 million people worldwide will be diagnosed with gastric cancer in 2018 [1]. It is the fifth most common cancer, accounting for 5–6% of all new cancer cases, but the third most prevalent cause of cancer mortality, contributing to 8–10% of cancer deaths [1, 2]. The highest incidence and mortality rates are typically observed in East Asia and Central and Eastern Europe [1].

The prognosis for gastric cancer is generally poor. Because it is asymptomatic in the early stages, more than half of gastric adenocarcinomas, which comprise 80–85% of gastric cancers, are diagnosed at an advanced stage (locally advanced or metastatic) [3]. Systemic therapy remains the mainstay of treatment for patients with locally advanced and metastatic disease, and has been shown to improve survival, organ function and performance status compared with best supportive care (BSC) [4]. Currently available treatment options include platinums, irinotecan, epirubicin, fluoropyrimidines, and taxanes. Two targeted agents, trastuzumab and ramucirumab, are also approved in a few countries for the treatment of advanced or metastatic gastric cancer.

Until recently, there was a lack of robust evidence to support the use of salvage chemotherapy for advanced patients who progress after first-line (1 L) treatment. A number of randomized controlled trials have demonstrated the efficacy and feasibility of second-line (2 L) treatment for these patients. Both irinotecan and docetaxel as monotherapy are associated with significantly improved survival versus placebo [5–8]. However, no significant clinical benefit was shown for combination therapy with irinotecan plus cisplatin compared with irinotecan alone [9, 10]. Use of ramucirumab in the 2 L setting has been shown to significantly improve survival, both as monotherapy versus placebo [11] and in combination with paclitaxel versus paclitaxel alone [12]. However, there was also evidence of increased grade 3 or worse leucopenia, neutropenia, anemia, hypertension, fatigue, and abdominal pain with the addition of ramucirumab to paclitaxel [12].

Despite these advances, therapeutic strategies for patients progressing after 1 L treatment are not clearly defined across clinical guidelines [13], and no consensus-driven standard of care has been defined. In the real-world setting, a number of studies have reported on 1 L treatment patterns and/or healthcare resource use (HCRU) in advanced or metastatic gastric cancer in Western countries [14–22]. However, evidence specific to patients who have progressed following 1 L treatment is limited. This article reports the results of GENERATE, an observational study on treatment patterns, HCRU and clinical outcomes of patients seen in routine clinical care who received 2 L therapy for advanced or metastatic gastric cancer. The study aims to improve the current understanding of the standard of care and the real-world patient outcomes in this setting using individual level data from four Western countries.

## Methods

### Objectives

The primary objectives of the GENERATE study were: 1) To describe the demographic and clinical characteristics of patients with metastatic or unresectable gastric or gastroesophageal junction (GEJ) cancer at initiation of 2 L anti-cancer systemic therapy after progression on previous therapy; 2) to describe treatment patterns in 2 L therapy in terms of drug regimen; 3) to describe HCRU during 2 L therapy, including management of cancer and adverse events (AEs), and biomarker testing. Secondary objectives were to assess real-world progression-free survival (rwPFS) and real-world overall survival (rwOS) from the start of 2 L therapy, both overall and by type of therapy, and to describe the factors associated with these outcomes.

### Study design

GENERATE was an observational, retrospective chart review study of a single pooled cohort which included patients with confirmed metastatic or unresectable gastric or GEJ adenocarcinoma who initiated 2 L drug therapy in Australia, Canada, Italy and the United Kingdom (UK). The investigators were medical oncologists, radiation oncologists and/or surgeons who specialized in gastrointestinal cancer management. Patients were identified starting from those who initiated 2 L therapy at the participating institutions in July 2015, and working backwards until an a priori target of 100 patients were identified for each country, or until January 2013. The index date was the day that the patient started 2 L therapy. Each patient’s chart was reviewed for the 12 months following the index date or until death, whichever occurred first; this was defined as the observation period.

Patients were included if they were aged ≥18 years at index date, had a diagnosis of metastatic or unresectable gastric or GEJ adenocarcinoma confirmed through histology and/or cytology, had a full medical history available at the participating site for the follow-up period, provided written informed consent (where required under national or institutional policies), were not participating in any interventional clinical trials during the follow-up period, and did not present with any other prior or concomitant malignancy, with the exception of basal cell carcinoma and squamous cell carcinoma of the skin, which were not excluded.

The study protocol was approved by the appropriate national or local ethics committee or institutional review board for each study site. Informed consent was collected for patients from Italy who were alive at the time of data abstraction. In the other countries, the need for informed consent was waived by the ethics committee due to the retrospective nature of the study and the minimal risk to patients. Demographic and clinical variables, gastric or GEJ cancer history and 1 L treatment were collected at the index date. Data that were collected for the entire follow-up period included details of 2 L and 3 L therapy like regimen, reason for discontinuation (if progression was stated, date of progression was defined as the date when treatment line was stopped), treatment outcomes (current or last available clinical status), and HCRU (hospitalizations, outpatient and emergency room visits, and laboratory and imaging tests performed).

All analyses were performed by country and for the overall study population. Since no hypothesis was tested, only descriptive statistics were employed. Additionally, Kaplan-Meier curves were used to present rwPFS and rwOS, and a multivariate model was used to explore the associations of covariates with rwPFS and rwOS. Patients with missing ECOG performance status were excluded from the final model.

## Results

### Baseline characteristics and 1 L treatment

Overall, 280 patients were included in the study: 34 from Australia (12.2%, from 6 sites), 100 from Canada (37.5%, from 6 sites), 84 from Italy (30.0%, from 8 sites), and 62 from the UK (22.1%, from 5 sites). Of these, 60 completed the 12-month observation period, 199 died before the end of the observation period, and 21 (of whom 14 were from Italy) were lost to follow-up. The majority of patients were male (68.9%), and Caucasian (84.9% of those with ethnicity recorded), and mean age was 60.9 (±12.0) years.

Almost all patients (93.6%) had received combination chemotherapy as 1 L treatment, of whom 67.9% had received triplet chemotherapy, most commonly anthracycline added to a fluoropyrimidine + platinum doublet. Approximately three-quarters (76.1%) of patients had undergone human epidermal growth factor receptor 2 (HER2) testing, of whom approximately one in five (22.0%) tested positive, and the addition of the anti-HER2 agent trastuzumab to the 1 L chemotherapy regimen was reported for 65.2% of these patients. Additional details regarding baseline patient characteristics, biomarker testing and 1 L treatment are shown in Table 1.

**Table 1:** Baseline sociodemographic and clinical characteristics, and 1 L treatment

	Australia (*N* = 34)	Canada (*N* = 100)	Italy (*N* = 84)	UK (*N* = 62)	Overall (*N* = 280)
**General demographic and clinical characteristics**					
Male gender, n (%)	22 (64.7%)	68 (68.0%)	61 (72.6%)	42 (67.7%)	193 (68.9%)
Age (years)					
Mean (SD)	65.7 (10.5)	60.7 (11.0)	60.6 (11.9)	58.8 (14.0)	60.9 (12.0)
Range	42–83	33–85	34–83	26–81	26–85
Ethnicity, n (%)					
Caucasian	32 (94.1%)	37 (60.7%)	84 (100.0%)	44 (83.0%)	197 (84.9%)
Asian	2 (5.9%)	11 (18.0%)	-	4 (7.5%)	17 (7.3%)
Other	-	13 (21.3%)	-	5 (9.4%)	18 (7.8%)
Unknown/missing	-	39	-	9	48
Smoking status, n (%)					
Current smoker	6 (23.1%)	13 (13.7%)	11 (14.5%)	7 (14.6%)	37 (15.1%)
Ex-smoker	9 (34.6%)	38 (40.0%)	37 (48.7%)	22 (45.8%)	106 (43.3%)
Non-smoker	11 (42.3%)	44 (46.3%)	28 (36.8%)	19 (39.6%)	102 (41.6%)
Unknown/missing	8	5	8	14	35
Alcohol consumption, n (%)^a^					
Heavy	1 (4.8%)	6 (6.7%)	2 (3.5%)	3 (7.0%)	12 (5.7%)
Occasional	11 (52.4%)	53 (59.6%)	37 (64.9%)	31 (72.1%)	132 (62.9%)
Abstinent	9 (42.9%)	30 (33.7%)	18 (31.6%)	9 (20.9%)	66 (31.4%)
Unknown/missing	13	11	27	19	70
Comorbidities, n (%)^b^					
None	8 (23.5%)	31 (31.0%)	42 (50.0%)	21 (33.9%)	102 (36.4%)
Hypertension	13 (38.2%)	29 (29.0%)	23 (27.4%)	24 (38.7%)	89 (31.8%)
Diabetes mellitus	4 (11.8%)	12 (12.0%)	11 (13.1%)	9 (14.5%)	36 (12.9%)
Dyslipidemia	7 (20.6%)	14 (14.0%)	6 (7.1%)	6 (9.7%)	33 (11.8%)
GI disease (other than gastric or GEJ cancer)	3 (8.8%)	11 (11.0%)	6 (7.1%)	3 (4.8%)	23 (8.2%)
Cardiovascular disease	5 (14.7%)	13 (13.0%)	7 (8.3%)	5 (8.1%)	30 (10.7%)
**Disease history and characteristics**					
Time since diagnosis (months), median (range)	12.01 (5.09–43.41)	7.79 (1.64–112.88)	9.91 (2.83–102.83)	9.76 (2.33–61.78)	9.40 (1.64–112.88)
Location of metastasis, n (%)^c^					
None	2 (5.9%)	6 (6.0%)	3 (3.6%)	3 (4.8%)	14 (5.0%)
Peritoneal	10 (29.4%)	34 (34.0%)	32 (38.1%)	18 (29.0%)	94 (33.6%)
Liver	21 (61.8%)	40 (40.0%)	33 (39.3%)	24 (38.7%)	118 (42.1%)
Bone	3 (8.8%)	13 (13.0%)	11 (13.1%)	5 (8.1%)	32 (11.4%)
Lung	7 (20.6%)	23 (23.0%)	20 (23.8%)	9 (14.5%)	59 (21.1%)
Other	11 (32.4%)	46 (46.0%)	30 (35.7%)	26 (41.9%)	113 (40.4%)
Number of metastatic locations, n (%)					
1	14 (43.8%)	39 (41.5%)	46 (56.8%)	31 (52.5%)	130 (48.9%)
2	8 (25.0%)	30 (31.9%)	14 (17.3%)	14 (23.7%)	66 (24.8%)
3	6 (18.8%)	14 (14.9%)	14 (17.3%)	7 (11.9%)	41 (15.4%)
4+	4 (12.5%)	11 (11.7%)	7 (8.6%)	7 (11.9%)	29 (10.9%)
ECOG PS at initiation of 2 L treatment, n (%)					
Unknown	9 (26.5%)	32 (32.0%)	9 (10.7%)	5 (8.1%)	55 (19.6%)
0	7 (28.0%)	7 (10.3%)	19 (25.3%)	12 (21.1%)	45 (20.0%)
1	17 (68.0%)	41 (60.3%)	45 (60.0%)	38 (66.7%)	141 (62.7%)
2	1 (4.0%)	18 (26.5%)	11 (14.7%)	5 (8.8%)	35 (15.6%)
3	-	2 (2.9%)	-	2 (3.5%)	4 (1.8%)
HER2 test, n (%)					
Not performed	20 (58.8%)	28 (28.0%)	14 (16.7%)	5 (8.1%)	67 (23.9%)
Performed	14 (41.2%)	72 (72.0%)	70 (83.3%)	57 (91.9%)	213 (76.1%)
Performed prior to 1 L initiation	8 (61.5%)	50 (76.9%)	60 (87.0%)	46 (85.2%)	164 (81.6%)
Performed later in treatment	5 (38.5%)	15 (23.1%)	9 (13.0%)	8 (14.8%)	37 (18.4%)
HER2 test positive^d^	3 (21.4%)	17 (24.6%)	15 (21.4%)	11 (19.6%)	46 (22.0%)
HER2 test negative^d^	11 (78.6%)	52 (75.4%)	55 (78.6%)	45 (80.4%)	163 (78.0%)
HER2 test result not available	-	3	-	1	4
History of surgery^e^	8 (24.2%)	15 (15.5%)	15 (18.3%)	4 (6.6%)	42 (15.4%)
History of radiotherapy^f^	4 (12.9%)	25 (25.5%)	2 (2.5%)	8 (13.3%)	39 (14.5%)
1 L anti-cancer drug therapy, n (%)^g^					
Monotherapy	3 (8.8%)	3 (3.0%)	9 (10.7%)	3 (4.8%)	18 (6.4%)
Fluoropyrimidine	1 (33.3%)	3 (100.0%)	4 (44.4%)	-	8 (44.4%)
Taxane	1 (33.3%)	-	4 (44.4%)	3 (100.0%)	8 (44.4%)
Platinum	-	-	1 (11.1%)	-	1 (5.6%)
Other monotherapy	1 (33.3%)	-	-	-	1 (5.6%)
Combination therapy	31 (91.2%)	97 (97.0%)	75 (89.3%)	59 (95.2%)	262 (93.6%)
Doublet chemotherapy	7 (22.6%)	25 (25.8%)	30 (40.0%)	12 (20.3%)	74 (28.2%)
Fluoropyrimidine + platinum	7 (22.6%)	22 (22.7%)	25 (33.3%)	11 (18.6%)	65 (24.8%)
Fluoropyrimidine + Irinotecan	-	1 (1.0%)	3 (4.0%)	-	4 (1.5%)
Other doublet	-	2 (2.1%)	2 (2.7%)	1 (1.7%)	5 (1.9%)
Triplet chemotherapy	22 (71.0%)	71 (73.2%)	42 (56.0%)	43 (72.9%)	178 (67.9%)
Fluoropyrimidine + platinum + anthracycline	21 (67.7%)	64 (66.0%)	18 (24.0%)	31 (52.5%)	134 (51.1%)
Fluoropyrimidine + platinum + other	1 (3.2%)	7 (7.2%)	23 (30.7%)	12 (20.3%)	43 (16.4%)
Other triplet	-	-	1 (1.3%)	-	1 (0.4%)

^a^ Heavy: > 3 (women) or > 4 (men) standard drinks/day; Occasional: < 3 or < 4 standard drinks/day, respectively; Abstinent = never drinks alcohol

^b^ Only comorbidities present in ≥5% of the overall study population are listed

^c^ Multiple responses were allowed

^d^ Out of patients who had test; information on test was missing/unkown in 12 patients (7 from Canada, 1–3 from other countries), result was missing/unknown in 3 patients from Canada and 1 from the UK

^e^ Surgical treatment of gastric/GEJ cancer; data were missing/unknown in 7 patients (1–3 in each country)

^f^ Includes neoadjuvant and palliative radiotherapy; data were missing/unknown in 11 patients (2–4 in each country)

^g^ Percentages for individual regimens are out of number of patients who received monotherapy or combination therapy, respectively

*1 L* first line, *ECOG* Eastern Cooperative Oncology Group, *GEJ* gastroesophageal junction, *GI* gastrointestinal, *HER2* human epidermal growth factor receptor 2, *SD* standard deviation

### 2 L treatment patterns

All patients initiated 2 L therapy, given that this was an inclusion criterion for the study. The treatments used are shown in Table 2; specific drug combinations for 2 L and 3 L therapy are described in greater detail in the supplementary material (Tables s1 and s2). Approximately half of patients (51.8%) received monotherapy, with no major differences in this proportion between countries. Taxanes (paclitaxel or docetaxel) were the most common monotherapy agents, used by 69.0% of all patients receiving monotherapy. However, there were considerable differences between countries: in the UK, 97.4% of monotherapy patients received taxanes, compared with only half in Australia and Canada. Irinotecan was used in 43.8% of Canadian monotherapy patients and 30.0% of those in Australia, but its use was limited in Italy and the UK. Few patients in any country received other monotherapies.

**Table 2:** 2 L chemotherapy

Therapy, n (%)^a^	Australia (*N* = 34)	Canada (*N* = 100)	Italy (*N* = 84)	UK (*N* = 62)	Overall (*N* = 280)
Monotherapy	20 (58.8%)	48 (48.0%)	38 (45.2%)	39 (62.9%)	145 (51.8%)
Combination therapy^b^	14 (41.2%)	52 (52.0%)	46 (54.8%)	23 (37.1%)	135 (48.2%)
Doublet chemotherapy	10 (71.4%)	39 (75.0%)	38 (82.6%)	15 (65.2%)	102 (75.6%)
Triplet chemotherapy	4 (28.6%)	13 (25.0%)	8 (17.4%)	7 (30.4%)	32 (23.7%)
Monotherapy^c^					
Taxanes	10 (50.0%)	23 (47.9%)	29 (76.3%)	38 (97.4%)	100 (69.0%)
Irinotecan	6 (30.0%)	21 (43.8%)	4 (10.5%)	1 (2.6%)	32 (22.1%)
Fluoropyrimidines	2 (10.0%)	3 (6.3%)	1 (2.6%)	-	6 (4.1%)
Ramucirumab	-	1 (2.1%)	3 (7.9%)	-	4 (2.8%)
Platinum	1 (5.0%)	-	1 (2.6%)	-	2 (1.4%)
Other monotherapy	1 (5.0%)	-	-	-	1 (0.7%)
Doublet chemotherapy^c^					
Fluoropyrimidine + irinotecan	2 (14.3%)	25 (48.1%)	18 (39.1%)	-	45 (33.3%)
Fluoropyrimidine + platinum	5 (35.7%)	6 (11.5%)	4 (8.7%)	9 (39.1%)	24 (17.8%)
Taxanes + ramucirumab	-	2 (3.8%)	10 (21.7%)	1 (4.3%)	13 (9.6%)
Taxanes + trastuzumab	-	-	3 (6.5%)	2 (8.7%)	5 (3.7%)
Other doublet	3 (21.4%)	6 (11.5%)	3 (6.5%)	3 (13.0%)	15 (11.1%)
Triplet chemotherapy^c^					
Fluoropyrimidine + platinum + anthracycline	4 (28.6%)	5 (9.6%)	3 (6.5%)	3 (13.0%)	15 (11.1%)
Fluoropyrimidine + platinum + other	-	8 (15.4%)	5 (10.9%)	2 (8.7%)	15 (11.1%)
Other triplet	-	-	-	2 (8.7%)	2 (1.5%)

^a^ Specific drugs used are tabulated in the supplementary material

^b^ Percentages receiving doublet and triplet are out of number of patients who received combination therapy

^c^ Percentages for individual regimens are out of number of patients who received monotherapy or combination therapy, respectively

There was more heterogeneity in the type of combination regimens used. Doublet chemotherapy was used in 36.4% of patients overall and 75.6% of patients receiving combination therapy. A third of patients on combination chemotherapy received fluoropyrimidine + irinotecan, and 17.8% received fluoropyrimidine + platinum. A small proportion received a taxane in combination with a novel agent (ramucirumab in 9.6% of patients receiving combination therapy and trastuzumab in 3.7%); nearly all of these cases were those treated in Italy. Almost a quarter of patients who received combination therapy (23.7%) were treated with triplet chemotherapy; this was almost always based on fluoropyrimidine + platinum, with either anthracycline or another agent added.

### 3 L treatment patterns

Overall, fewer than a third of patients (29.6%) received 3 L chemotherapy, and the proportion was largely similar between countries (Table 3). Roughly equal proportions received monotherapy and combination therapy, except in Canada where combination therapy was less common in 3 L. As in 2 L, taxanes were the most commonly used monotherapy, followed by irinotecan. Few patients received monotherapy with novel agents. Most patients who had combination therapy received a doublet, most commonly fluoropyrimidines in combination with irinotecan or platinum; triplet therapy was uncommon.

**Table 3:** 3 L chemotherapy

Therapy, n (%)^a^	Australia (N = 9)	Canada (*N* = 37)	Italy (*N* = 19)	UK (*N* = 18)	Overall (*N* = 83)
Monotherapy	4 (44.4%)	28 (75.7%)	10 (52.6%)	10 (55.6%)	52 (62.7%)
Combination therapy^b^	5 (55.6%)	9 (24.3%)	9 (47.4%)	8 (44.4%)	31 (37.3%)
Doublet chemotherapy	5 (100.0%)	7 (77.8%)	8 (88.9%)	6 (85.7%)	26 (86.7%)
Triplet chemotherapy	-	2 (22.2%)	1 (11.1%)	1 (14.3%)	4 (13.3%)
Missing	-	-	-	1	1
Monotherapy^c^					
Taxanes	3 (75.0%)	18 (64.3%)	8 (80.0%)	7 (70.0%)	36 (69.2%)
Irinotecan	1 (25.0%)	8 (28.6%)	-	1 (10.0%)	10 (19.2%)
Ramucirumab	-	1 (3.6%)	1 (10.0%)	1 (10.0%)	3 (5.8%)
Fluoropyrimidines	-	1 (3.6%)	1 (10.0%)	-	2 (3.8%)
Other monotherapy	-	-	-	1 (10.0%)	1 (1.9%)
Doublet chemotherapy^c^					
Fluoropyrimidine + irinotecan	5 (100.0%)	2 (22.2%)	4 (44.4%)	5 (71.4%)	16 (53.3%)
Fluoropyrimidine + platinum	-	3 (33.3%)	3 (33.3%)	-	6 (20.0%)
Taxanes + ramucirumab	-	1 (11.1%)	1 (11.1%)	1 (14.3%)	3 (10.0%)
Other doublet	-	1 (11.1%)	-	-	1 (3.3%)
Triplet chemotherapy^c^					
Fluoropyrimidine + platinum + anthracycline	-	1 (11.1%)	-	-	1 (3.3%)
Fluoropyrimidine + platinum + other	-	1 (11.1%)	1 (11.1%)	-	2 (6.7%)
Other triplet	-	-	-	1 (14.3%)	1 (3.3%)

^a^ Specific drugs used are tabulated in the supplementary material

^b^ Percentages receiving doublet and triplet are out of number of patients who received combination therapy

^c^ Percentages for individual regimens are out of number of patients who received monotherapy, doublet or triplet therapy, respectively

**Fig. 1:**
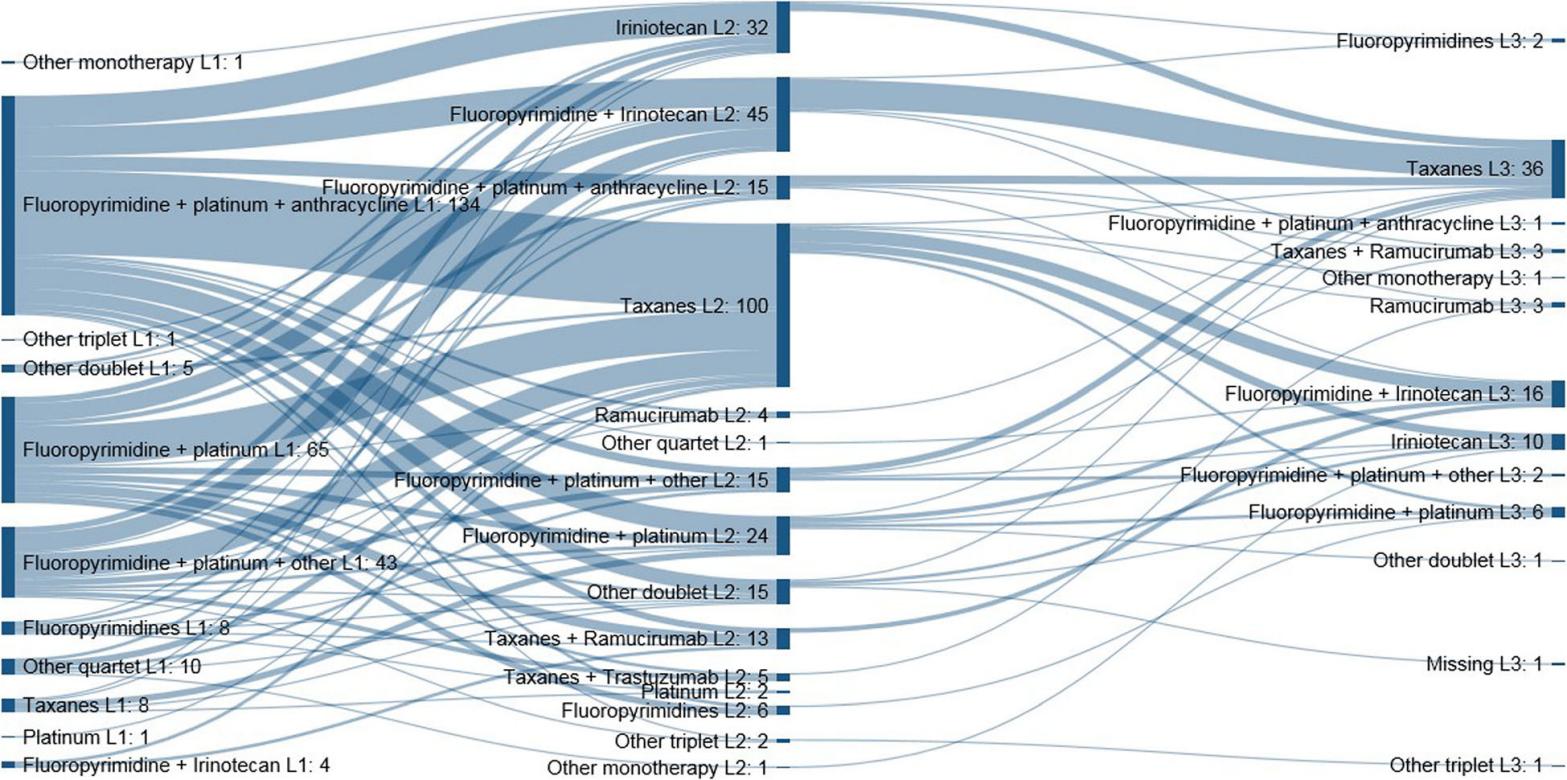
Sankey Diagram of most common therapies prescribed in 1 L, 2 L and 3 L treatment

Patients’ transition from 1 L to 2 L and 3 L of treatment is shown in Fig. 1 as a Sankey Diagram of the most common therapies that were used in the next line following specific therapies in the previous line. There was a general trend for patients to proceed from a fluoropyrimidine/platinum/anthracycline triplet or a fluoropyrimidine/platinum doublet in 1 L, to 2 L treatment based on irinotecan or taxanes as monotherapy or a fluoropyrimidine/irinotecan doublet, followed by 3 L treatment consisting of taxane monotherapy or fluoropyrimidine/irinotecan doublet regimens.

### Real-world treatment outcomes

Median rwPFS for patients initiating 2 L treatment was estimated to be 3.09 (95% CI: 2.76–3.68) months from the date of initiation of therapy. The estimated probability of rwPFS at 3, 6, 9 and 12 months was 51, 27, 14 and 8%, respectively. Median (95% CI) rwOS was estimated to be 6.54 (5.29–7.76) months, and the estimated probability of rwOS at 6, 12, 18 and 24 months after treatment initiation was 53, 26, 15 and 5%, respectively.

**Fig. 2:**
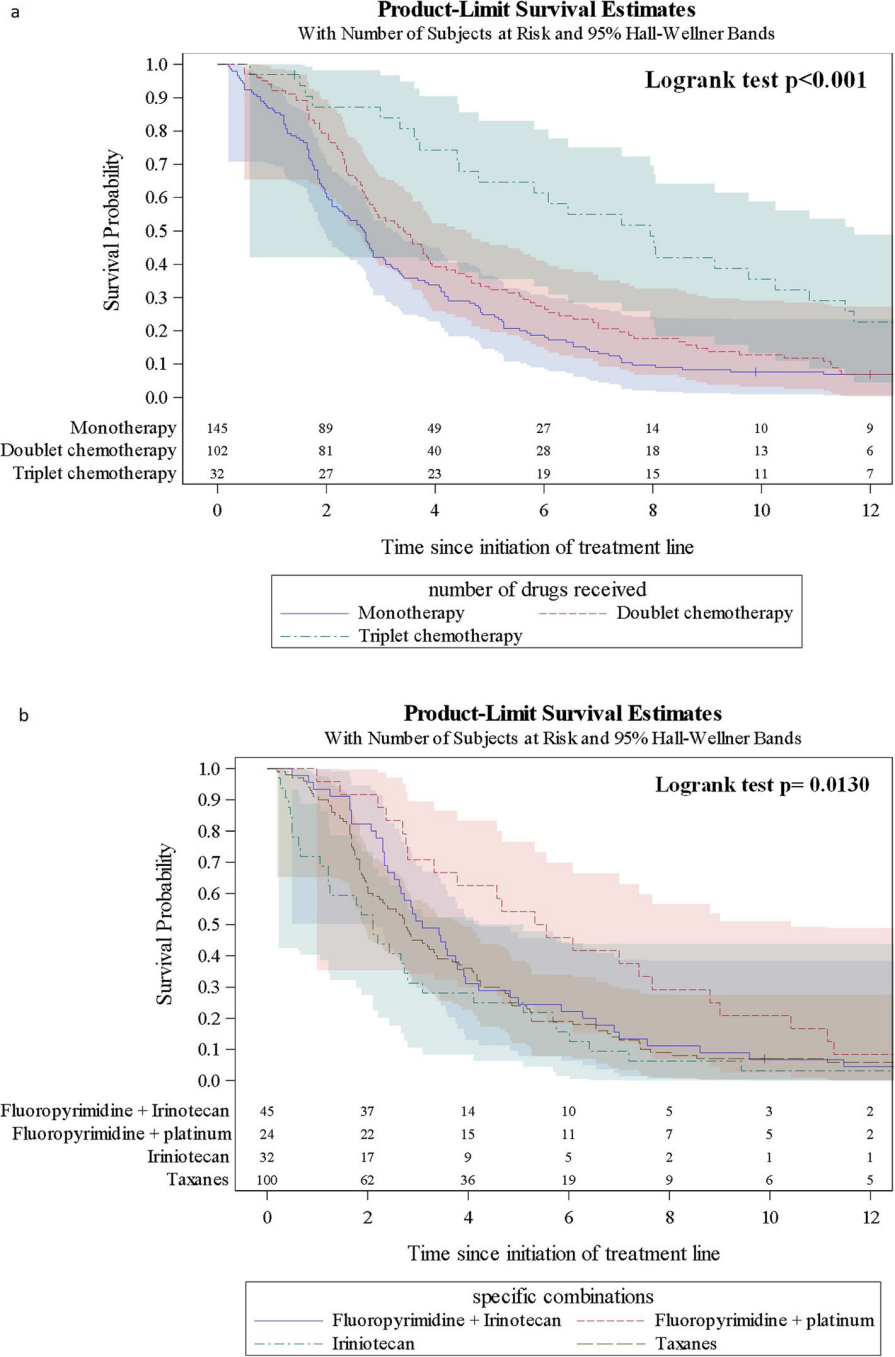
Progression-free survival from initiation of 2 L treatment. **a** PFS with monotherapy, doublet therapy, triplet therapy, and overall; **b** PFS with taxane monotherapy, irinotecan monotherapy, fluoropyrimidine + irinotecan, and fluoropyrimidine + platinum

**Fig. 3:**
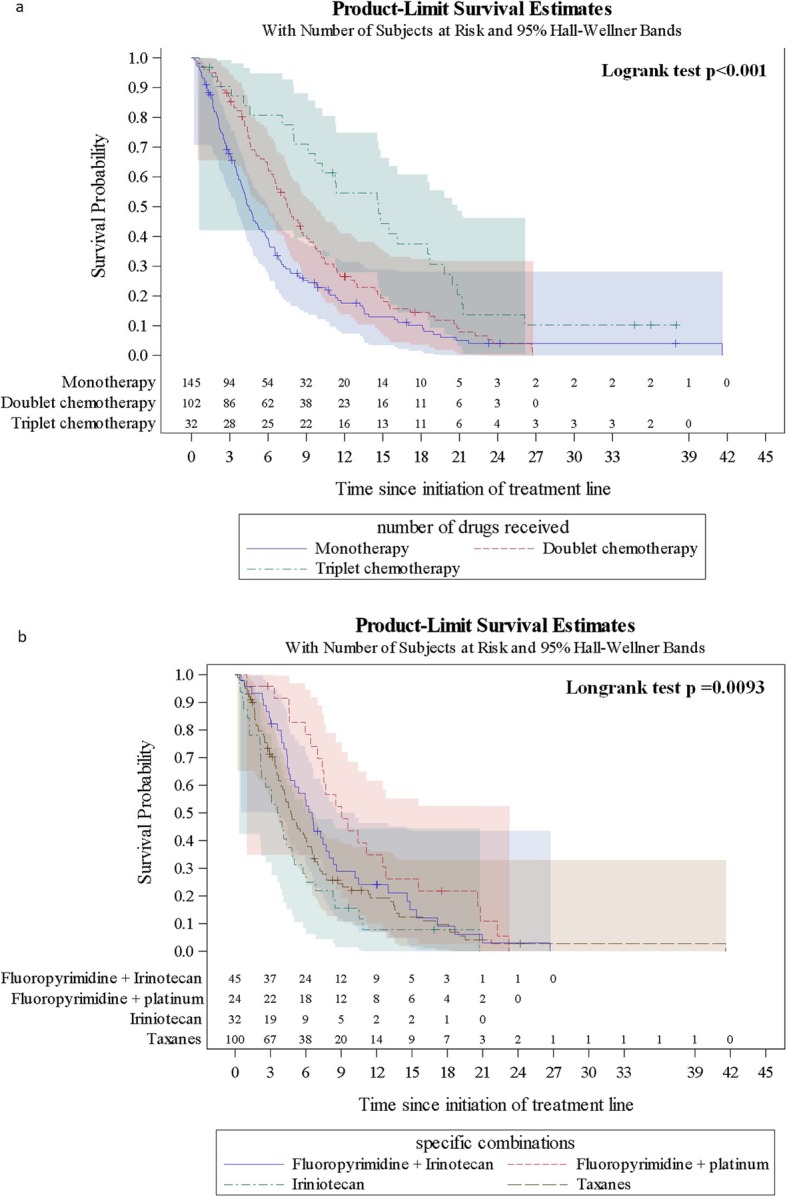
Overall survival from initiation of 2 L treatment. **a** OS with monotherapy, doublet therapy, triplet therapy, and overall; **b** OS with taxane monotherapy, irinotecan monotherapy, fluoropyrimidine + irinotecan, fluoropyrimidine + platinum, and overall

When evaluated according to treatment regimen, both rwPFS and rwOS showed notable differences (see Figs. 2 and 3). Median rwPFS increased from 2.69 (95% CI 2.11–2.86) months with monotherapy to 3.43 (95% CI 2.73–3.91) months with doublet chemotherapy (statistical significance not reached), and 7.95 (95% CI 4.43–10.25) with triplet chemotherapy (*p* < 0.001, Log-rank test). Similarly, median rwOS increased from 4.50 (95% CI 3.81–5.52) months with monotherapy to 7.53 (95% CI 6.41–8.74) months with doublet chemotherapy and 14.66 (95% CI 9.13–18.56) with triplet chemotherapy (p < 0.001, Log-rank test).

In terms of survival with individual 2 L treatment regimens, taxane monotherapy and irinotecan monotherapy showed similar rwPFS (median 2.74 [95% CI 2.00–3.35] and 2.10 [95% CI 1.05–2.79] months, respectively) (Fig. 2), but rwOS with taxanes was slightly longer (median 4.73 [95% CI 3.88–6.05] versus 3.70 [95% CI 2.17–5.03]) (Fig. 3). Of the most common doublet therapies, PFS was numerically longer with fluoropyrimidine + platinum (5.44 [95% CI 2.76–7.66] months) than with fluoropyrimidine + irinotecan (3.09 [95% CI 2.53–3.78] months). This translated into numerically longer rwOS with fluoropyrimidine + platinum (9.04 [7.00–12.52] versus 6.54 [4.57–7.99] months).

In the multivariate analysis of rwOS, use of combination therapy in 2 L was associated with a trend for longer survival compared with monotherapy (HR for death 0.738 [95% CI 0.51–1.08], *p* = 0.114). Factors associated with shorter survival were ECOG performance status > 1 at 2 L treatment initiation (HR 1.50 [95% CI 0.94–2.40], *p* = 0.090), and presence of more than one metastatic location (HR 1.58 [95% CI 1.07–2.32], *p* = 0.010). However, only the association with number of metastatic locations reached statistical significance. Presence of > 1 metastatic location was also significantly associated with shorter PFS (HR 1.51 [95% CI 1.048–2.172], *p* = 0.027). There were non-significant associations between female gender and shorter rwPFS, and combination therapy and longer rwPFS. Results for the multivariate analyses are shown in the supplementary material (Table s3 and Table s4).

### Healthcare resource use

The number and percentage of patients using each healthcare resource during 2 L treatment, regardless of reason for the use, is shown in Table 4. Average follow-up period was 6.6 months. Only a minority (26.8%) of patients were hospitalized (hospitalization may have been same-day), with hospitalization least likely in Italy (16.7%, versus 30.0–32.4% elsewhere). Intensive care unit (ICU) stays were rare. Almost all patients (94.6%) received concomitant medications. Laboratory tests and imaging studies were performed for 93.2 and 70.4% of patients, respectively. Patients in Italy were markedly less likely to undergo imaging tests (44.0%, versus 76.5–90.3% elsewhere).

**Table 4:** Number and percentage of patients using each healthcare resource during 2 L treatment

Resource, n (%)^a^	Australia (*N* = 34)	Canada (*N* = 100)	Italy (*N* = 84)	UK (*N* = 62)	Overall (*N* = 280)
Hospitalization/inpatient stay	11 (32.4%)	30 (30.0%)	14 (16.7%)	20 (32.3%)	75 (26.8%)
ICU stay	-	2 (2.0%)	-	-	2 (0.7%)
Emergency room visit	3 (8.8%)	19 (19.0%)	2 (2.4%)	7 (11.3%)	31 (11.1%)
Outpatient (visit for follow-up)	21 (61.8%)	27 (27.0%)	51 (60.7%)	50 (80.6%)	149 (53.2%)
Concomitant medication	30 (88.2%)	91 (91.0%)	84 (100.0%)	60 (96.8%)	265 (94.6%)
Laboratory tests	27 (79.4%)	95 (95.0%)	78 (92.9%)	61 (98.4%)	261 (93.2%)
Blood cell count	27 (79.4%)	94 (94.0%)	78 (92.9%)	61 (98.4%)	260 (92.9%)
Biochemistry test	27 (79.4%)	92 (92.0%)	78 (92.9%)	61 (98.4%)	258 (92.1%)
Liver function test	27 (79.4%)	92 (92.0%)	78 (92.9%)	57 (91.9%)	254 (90.7%)
Renal function test	27 (79.4%)	94 (94.0%)	77 (91.7%)	56 (90.3%)	254 (90.7%)
Blood pressure reading	26 (76.5%)	53 (53.0%)	58 (69.0%)	49 (79.0%)	186 (66.4%)
Electrocardiogram	7 (20.6%)	18 (18.0%)	21 (25.0%)	14 (22.6%)	60 (21.4%)
Imaging tests	26 (76.5%)	78 (78.0%)	37 (44.0%)	56 (90.3%)	197 (70.4%)
*X-ray*	11 (32.4%)	44 (44.0%)	17 (20.2%)	10 (16.1%)	82 (29.3%)
*Ultrasound*	8 (23.5%)	24 (24.0%)	5 (6.0%)	9 (14.5%)	46 (16.4%)
CT	25 (73.5%)	60 (60.0%)	27 (32.1%)	54 (87.1%)	166 (59.3%)
MRI scan	1 (2.9%)	5 (5.0%)	1 (1.2%)	2 (3.2%)	9 (3.2%)
PET scan	-	1 (1.0%)	-	1 (1.6%)	2 (0.7%)
Fluorodeoxyglucose-PET-CT scan	5 (14.7%)	2 (2.0%)	3 (3.6%)	1 (1.6%)	11 (3.9%)
Endoscopy	2 (5.9%)	7 (7.0%)	2 (2.4%)	5 (8.1%)	16 (5.7%)
Colonoscopy	-	1 (1.0%)	1 (1.2%)	-	2 (0.7%)

^a^ number and percentage of patients for whom the resource has been used; denominator is the total number of patients

*CT* Computed tomography, *ICU* intensive care unit, *MRI* Magnetic resonance imaging, *PET* Positron emission tomography

## Discussion

The GENERATE study provides a detailed description of patient characteristics, treatment patterns, HCRU and survival outcomes for real-world patients initiating 2 L treatment for advanced or metastatic gastric or GEJ adenocarcinoma who were seen in routine clinical practice across four different Western countries.

The sociodemographic and clinical characteristics of patients included in GENERATE were broadly comparable with patients in randomized controlled trials (RCTs). In terms of age, patients included in GENERATE were similar to participants included in the most recent RCTs evaluating 2 L treatment [7, 8, 10–12, 23, 24]. Patients included in earlier RCTs, which were the first to provide evidence on the efficacy of 2 L treatment, were slightly younger, with median ages ranging between 55 and 56 years [5, 6].

Only 7.3% of patients for whom ethnicity was reported were Asian, whereas a number of RCTs have had predominant Asian populations [6, 8–10, 23, 24]. In contrast to most RCTs, in which all or the great majority of patients are of good performance status (PS; ECOG 0 or 1) [6, 8–12, 23, 24], approximately 16% of patients in GENERATE for whom PS data were available had ECOG PS of 2, and 2% had PS of 3.

Patients in GENERATE were also less likely to have had prior surgery: 15.4% had surgery for their cancer, compared with 21–58% in 2 L RCTs [5, 6, 8, 9, 12, 23, 24]. This might be because they tended to be older and less fit than patients enrolled in RCTs.

Drugs prescribed as 1 L chemotherapy to patients included in the GENERATE study were also consistent with those reported for patients included in RCTs, with platinum/fluoropyrimidine and taxane/fluoropyrimidine combinations being the most commonly prescribed. However, there were differences in the proportion of patients who had received triplet vs doublet 1 L regimens. Those in RCT settings were more likely to have received doublet regimens (60–100% of patients) than triplet ones (6–26%) [5, 6, 8, 9, 11, 12], whereas almost two-thirds of patients in GENERATE had received fluoropyrimidine/platinum triplet combinations as 1 L therapy, rather than a doublet. This is unsurprising in the light of evidence demonstrating the significant benefit from addition of an anthracycline to the fluoropyrimidine/platinum regimen [25].

Treatment outcomes reported in GENERATE were also broadly comparable to those reported in RCTs evaluating 2 L treatments, with modest PFS and OS. For instance, median PFS and OS with irinotecan in an RCT of irinotecan versus BSC were 2.6 and 4.0 months, respectively [5], which are similar to those reported here for irinotecan monotherapy (rwPFS 2.10 months and rwOS 3.70 months). A phase 3 RCT comparing docetaxel with BSC reported a median OS of 5.2 months for docetaxel [7], which is also consistent with the estimated rwOS of 4.7 months for patients treated with taxane monotherapy reported in GENERATE.

More recently, several RCTs have reported efficacy outcomes for PD-1 and PD-1L-targeted immunotherapies in second and subsequent treatment lines. In a phase III RCT comparing pembrolizumab with paclitaxel in 2 L, patients treated with pembrolizumab presented with a median PFS and OS 1.5 and 9.1 months, respectively [26]. In a phase I/II RCT comparing nivolumab monotherapy with two combinations of nivolumab and ipilimumab in patients having received at minimum a first line of treatment, the median PFS and OS for nivolumab monotherapy was 1.4 and 6.2 months, respectively, and ranged between 1.4–1.6 and 4.8–6.9 months, respectively, for the two combination therapy regimens [27]. In the 3 L setting, the median PFS and OS reported in a phase III RCT that compared avelumab vs physician’s choice of chemotherapy was 1.4 and 4.6 months, respectively [28]. These findings place immunotherapy as a potential good alternative for advanced GEJ cancer patients but further studies are awaited to help define these as regular treatment choices for these patients.

Similarly, other novel therapies such as apatinib (rivoceranib) or trifluridine/tipiracil have been recently evaluated in the 3 L setting. In a double-blind phase III RCT conducted in China comparing apatinib vs. placebo in patients who had failed to at least two previous lines of chemotherapy, the median PFS and OS for apatinib were 2.6 and 6.5 months, respectively [29]. Very recently, results of a global phase III RCT also comparing apatinib vs. placebo indicated that apatinib patents had a median PFS and OS of 2.8 and 5.8 months, respectively although OS was not significantly improved for patients receiving apatinib in 3 L therapy [30]. In another multi-country doble-blind phase III RCT, that evaluated trifluridine/tipiracil plus BSC vs. placebo plus BSC, the median PFS and OS were 2.0 and 5.7 months, respectively [31]. These results show the potential to widen the scope of alternative tretments for patients with advanced GEJ cancer, although further studies are warranted to evaluate the potential use of these drugs in the 2 L setting.

GENERATE found that approximately half of all patients (51.8%) had monotherapy in 2 L treatment, with taxanes (used by 69.0% of monotherapy patients) and irinotecan (22.1%) being the most commonly prescribed monotherapies. This is consistent with evidence showing increased OS with these agents compared with BSC [5–7]. Use of ramucirumab was infrequent, reported in 6 patients as monotherapy (4.1% of monotherapy patients) and 13 patients (9.6% of combination therapy patients) in combination with taxanes. Almost all of this use was in Italy, as ramucirumab had not yet been approved in Canada or Australia during the study period (index date between January 2013 and July 2015), and although approved by the EMA in December 2014 [32, 33] it was not recommended by NICE in the UK. Use of trastuzumab in 2 L was also infrequent (16 patients in total, most in combination with taxanes, or as triplet therapy with platinum and fluoropyrimidines).

The findings from GENERATE on treatment patterns are generally similar to those of previous studies evaluating routine clinical practices, although methodological differences between the studies make direct comparisons difficult [14–18, 22]. A retrospective analysis by Fanatto et al. of 300 patients with advanced or metastatic gastric cancer in Italy who had received at least three chemotherapy regimens between 2000 and 2015 found that for 2 L therapy, 37.3, 48.7 and 9.7% of patients were treated with monotherapy, doublet and triplet regimens, respectively [15]. Fluoropyrimidines (52.7% of patients), taxanes (39.7%) and irinotecan (39.0%) were the most commonly prescribed chemotherapy agents in 2 L. [15] Liepa et al. retrospectively reported on 200 patients in the UK dignosed with advanced or metastatic gastric cancer between 2007 and 2012 and who had received 1 L treatment with a fluoropyrimidine/platinum regimen, 57 of whom received 2 L therapy [16]. The lack of a clearly defined standard of care was reflected in the fact that 21 unique 2 L regimens were reported by Liepa et al., with monotherapy being most common. The most commonly used agents were docetaxel (28% of patients), paclitaxel (11%), trastuzumab (9%), capecitabine (7%) and irinotecan (7%) [16]. In a retrospective study by Elsing et al. of 111 patients in Germany who initiated treatment between 2001 and 2011, the most frequently used 2 L treatment was irinotecan in combination with 5-FU (21% of patients), and 19% of patients received irinotecan monotherapy [22]; again, this was similar to the treatment patterns observed in GENERATE. However, Elsing et al. showed that taxane monotherapy was not prescribed as 2 L treatment, probably because the study included patients who began treatment before evidence on the efficacy of taxanes in 2 L therapy was published.

In terms of HCRU, the data from GENERATE will be useful to inform economic evaluations of 2 L treatments for advanced or metastatic gastric cancer, and complement the findings of other studies with different designs and study populations and in different healthcare systems [14, 16, 18, 34]. Overall, there was a mean observation period of 6.6 months and approximately a quarter of patients required hospitalization. Admission to ICU was reported in less than 1% of patients. Emergency room visits were required by over 10% of patients, whereas outpatient visits were reported by approximately half of patients. Almost all patients received concomitant medications and required laboratory tests, and a high percentage underwent imaging tests (70.4%). Thus, results from GENERATE indicate substantial resource use for this population.

The study by Liepa et al. [16] conducted in the UK reported that 63, 39 and 46% of patients required follow-up outpatient visits, emergency room visits and hospitalizations, respectively, but this was measured from the start of 1 L treatment, rather than the start of 2 L treatment as in GENERATE. In addition, Liepa et al. excluded outpatient visits related to chemotherapy administration. In GENERATE, more UK patients (80.6%) had outpatient visits than in Liepa et al., but fewer required emergency room visits (11.3%) and hospitalization (32.2%).

Karve et al. reported a retrospective analysis of the Surveillance, Epidemiology, and End Results (SEER)-Medicare-linked database that included 2583 patients diagnosed with locally advanced/unresectable or metastatic gastric cancer in the US between 2000 and 2007. Patients were aged ≥65 and had received 1 L treatment with fluoropyrimidine and/or a platinum chemotherapy agent [14]. In the Karve study, 33.9, 16.8 and 82.5% of patients who received 2 L treatment required hospitalizations, emergency room visits and outpatient visits, respectively. The proportions of patients requiring hospitalizations and emergency room visits were relatively similar to those observed in GENERATE (which were 26.8 and 11.1%, respectively), whereas the proportion requiring outpatient visits in GENERATE was much lower (53.2%). This may be explained by the longer observation period in the study conducted by Karve et al.

There are several strengths associated with the GENERATE study. Eligible patients had to have been followed at the participating site and have clinical chart data available for the entire observation period, thus enabling a comprehesive and longitudinal picture of treatment and HRCU during 2 L therapy. Detailed information on treatment patterns, HCRU and outcomes was collected for patients in whom the full medical history was available, from initiation of 2 L treatment and for a 12-month follow up, which would cover the whole treatment line in most cases. Participating physicians were all experienced in the management of gastric cancer.

A few study limitations need to be noted. The study was based on retrospective chart review, which may be associated with systematic under-recording of some types of information on the clinical charts. Secondly, assessment of disease progression was based exclusively on evaluation by the treating physician, and may have been influenced by local diagnostic practices and treatment standards. There may also have been inconsistencies in the time points at which disease progression was assessed. Thirdly, the study data were collected from a convenience sample of sites that routinely manage patients with advanced or metastatic gastric cancer, and may not be representative of all sites in the respective countries. Finally, the real burden of the management of advanced gastric cancer patients may be understimated, as the use of healthcare resources associated with palliative care was not specifically captured in this study.

## Conclusions

An increasing number of studies have shown 2 L treatment for advanced or metastatic gastric cancer to be effective and feasible. However, despite recent evidence, there are still no clear recommendations established across clinical guidelines to enable an understanding of current treatment patterns. To this end, the GENERATE study adds to the literature by providing insights on patient characteristics, treatment patterns, HCRU and clinical outcomes of real-world patients diagnosed with advanced or metastatic gastric or GEJ adenocarcinoma who initiated 2 L therapy between January 2013 and July 2015 across four different countries (Australia, Canada, Italy and the UK). Further work, including the collection of more recent data and the collection of data on palliative care, would further clarify contemporary treatment patterns in this setting and help understand the burden of lost lifes.

## Supplementary information

**Additional file 1: Table S1.** Drugs received in 2 L chemotherapy. **Table S2.** Drugs received in 3 L chemotherapy. **Table S3.** Overall survival multivariate analysis results (excluding patients with ECOG PS missing). **Table S4.** Progression-free survival multivariate analysis results (excluding patients with ECOG PS missing).

## Data Availability

The datasets used and analysed during the current study are available from the corresponding author on reasonable request.

## Abbreviations

1 L: First line
2 L: Second line
3 L: Third line
AE: Adverse event
BSC: best supportive care
ECOG: Eastern Cooperative Oncology Group Performance Status test
GEJ: Gastroesophageal junction
HCRU: Healthcare resource utilization
HER2: Human epidermal growth factor receptor 2
ICU: Intensive care unit
OS: Overall survival
PFS: Progression free survival
RCT: Randomized controlled trials
rwOS: Real-world overall survival
rwPFS: Real-world progression free survival
UK: United Kingdom
