# Supplementary material for: Real-world treatment patterns, healthcare resource use and clinical outcomes of patients receiving second line therapy for advanced or metastatic gastric cancer
Source: BMC Gastroenterol. 2020 May 5;20:133. doi: 10.1186/s12876-020-01232-z (PMC7201990; doi:10.1186/s12876-020-01232-z)
Supplement: Supplementary file 1 — Additional file 1: Table S1. Drugs received in 2 L chemotherapy. Table S2. Drugs received in 3 L chemotherapy. Table S3. Overall survival multivariate analysis results (excluding patients with ECOG PS missing). Table S4. Progression-free survival multivariate analysis results (excluding patients with ECOG PS missing). [file 12876_2020_1232_MOESM1_ESM.docx]

**Table s1 Drugs received in 2L chemotherapy**

|  | **Australia (N=34)** | **Canada (N=100)** | **Italy (N=84)** | **UK (N=62)** | **Overall (N=280)** |
| --- | --- | --- | --- | --- | --- |
| Monotherapy drugs^a^ | | | | | |
| PAC | 10 (50.0%) | 11 (22.9%) | 14 (36.8%) | 28 (71.8%) | 63 (43.4%) |
| DOCE |  | 12 (25.0%) | 15 (39.5%) | 10 (25.6%) | 37 (25.5%) |
| IRIN | 6 (30.0%) | 21 (43.8%) | 4 (10.5%) | 1 (2.6%) | 32 (22.1%) |
| CAPE | 2 (10.0%) | 3 (6.3%) |  |  | 5 (3.4%) |
| RAMU |  | 1 (2.1%) | 3 (7.9%) |  | 4 (2.8%) |
| CISP |  |  | 1 (2.6%) |  | 1 (0.7%) |
| 5-FU |  |  | 1 (2.6%) |  | 1 (0.7%) |
| GEMCI | 1 (5.0%) |  |  |  | 1 (0.7%) |
| OXAL | 1 (5.0%) |  |  |  | 1 (0.7%) |
| Combination therapy drugs^a^ | | | | | |
| 5-FU+IRIN | 2 (14.3%) | 21 (40.4%) | 18 (39.1%) |  | 41 (30.4%) |
| PAC+RAMU |  | 1 (1.9%) | 10 (21.7%) | 1 (4.3%) | 12 (8.9%) |
| CAPE+CISP+EPI | 1 (7.1%) | 4 (7.7%) | 2 (4.3%) | 1 (4.3%) | 8 (5.9%) |
| CAPE+CARB |  | 1 (1.9%) |  | 6 (26.1%) | 7 (5.2%) |
| CAPE+CISP+TRAST |  | 6 (11.5%) |  | 1 (4.3%) | 7 (5.2%) |
| CARB+PAC |  | 5 (9.6%) |  |  | 5 (3.7%) |
| 5-FU+OXAL | 2 (14.3%) |  | 2 (4.3%) | 1 (4.3%) | 5 (3.7%) |
| CAPE+CISP | 1 (7.1%) | 2 (3.8%) |  | 1 (4.3%) | 4 (3.0%) |
| CAPE+OXAL+EPI | 1 (7.1%) | 1 (1.9%) |  | 2 (8.7%) | 4 (3.0%) |
| CAPE+IRIN |  | 4 (7.7%) |  |  | 4 (3.0%) |
| 5-FU+CISP |  | 2 (3.8%) | 2 (4.3%) |  | 4 (3.0%) |
| CAPE+OXAL | 1 (7.1%) | 1 (1.9%) |  | 1 (4.3%) | 3 (2.2%) |
| 5-FU+CISP+EPI | 2 (14.3%) |  | 1 (2.2%) |  | 3 (2.2%) |
| 5-FU+OXAL+IRIN |  |  | 3 (6.5%) |  | 3 (2.2%) |
| PAC+TRAST |  |  | 1 (2.2%) | 2 (8.7%) | 3 (2.2%) |
| CAPE+DOCE+IRIN |  |  |  | 2 (8.7%) | 2 (1.5%) |
| CISP+PAC |  | 1 (1.9%) |  | 1 (4.3%) | 2 (1.5%) |
| DOCE+TRAST |  |  | 2 (4.3%) |  | 2 (1.5%) |
| CAPE+CARB+TRAST |  |  |  | 1 (4.3%) | 1 (0.7%) |
| CAPE+CISP+DOCE+EPI |  |  |  | 1 (4.3%) | 1 (0.7%) |
| 5-FU+CAPE+CISP+TRAST |  | 1 (1.9%) |  |  | 1 (0.7%) |
| CAPE+CYCLO |  |  | 1 (2.2%) |  | 1 (0.7%) |
| CAPE+DOCE |  |  | 1 (2.2%) |  | 1 (0.7%) |
| 5-FU+CAPE+OXAL |  |  | 1 (2.2%) |  | 1 (0.7%) |
| CAPE+LAPAT | 1 (7.1%) |  |  |  | 1 (0.7%) |
| CAPE+PAC | 1 (7.1%) |  |  |  | 1 (0.7%) |
| 5-FU+CARBO | 1 (7.1%) |  |  |  | 1 (0.7%) |
| 5-FU+CISP+DOCE |  |  | 1 (2.2%) |  | 1 (0.7%) |
| 5-FU+CISP+TRAST |  | 1 (1.9%) |  |  | 1 (0.7%) |
| CISP+TEGA |  |  |  | 1 (4.3%) | 1 (0.7%) |
| DOCE+PAC+RAMU |  | 1 (1.9%) |  |  | 1 (0.7%) |
| OXAL+EPI |  |  |  | 1 (4.3%) | 1 (0.7%) |
| EPI+PAC |  |  | 1 (2.2%) |  | 1 (0.7%) |
| 5-FU+TRAST | 1 (7.1%) |  |  |  | 1 (0.7%) |
| ^a^Percentages calculated out of total patients in monotherapy or total patients in combination therapy  5-FU: 5-Fluorouracil; CARB: carboplatin; CAPE: capecitabine; CISP: cisplatin; DOCE: docetaxel; EPI: epirubicin; IRIN: irinotecan; ONAR: onartuzumab; OXAL: oxaliplatin; PAC: paclitaxel; PL: placebo; REGO: regorafenib; RILOT: rilotumumab; TEGA: tegafur; TRAST: trastuzumab | | | | | |

**Table s2 Drugs received in 3L chemotherapy**

|  | **Australia (N=9)** | **Canada (N=37)** | **Italy (N=19)** | **UK (N=18)** | **Overall (N=83)** |
| --- | --- | --- | --- | --- | --- |
| Monotherapy drugs^a^ | | | | | |
| DOCE | 3 (75.0%) | 9 (32.1%) | 3 (30.0%) | 4 (40.0%) | 19 (36.5%) |
| PAC |  | 9 (32.1%) | 5 (50.0%) | 3 (30.0%) | 17 (32.7%) |
| IRIN | 1 (25.0%) | 8 (28.6%) |  | 1 (10.0%) | 10 (19.2%) |
| RAMU |  | 1 (3.6%) | 1 (10.0%) | 1 (10.0%) | 3 (5.8%) |
| CAPE |  |  | 1 (10.0%) |  | 1 (1.9%) |
| 5-FU |  | 1 (3.6%) |  |  | 1 (1.9%) |
| NIVO |  |  |  | 1 (10.0%) | 1 (1.9%) |
| Combination therapy drugs^a^ | | | | | |
| 5-FU+IRIN | 5 (100.0%) | 2 (22.2%) | 4 (44.4%) | 3 (42.9%) | 14 (46.7%) |
| 5-FU+OXAL |  | 1 (11.1%) | 3 (33.3%) |  | 4 (13.3%) |
| PAC+RAMU |  | 1 (11.1%) | 1 (11.1%) | 1 (14.3%) | 3 (10.0%) |
| CAPE+IRIN |  |  |  | 2 (28.6%) | 2 (6.7%) |
| CAPE+CISP |  | 1 (11.1%) |  |  | 1 (3.3%) |
| CAPE+CISP+EPI |  | 1 (11.1%) |  |  | 1 (3.3%) |
| CAPE+CISP+TRAST |  | 1 (11.1%) |  |  | 1 (3.3%) |
| 5-FU+CAPE+CISP |  | 1 (11.1%) |  |  | 1 (3.3%) |
| CAPE+TRAST |  | 1 (11.1%) |  |  | 1 (3.3%) |
| CAPE+IRIN+EPI |  |  |  | 1 (14.3%) | 1 (3.3%) |
| 5-FU+OXAL+IRIN |  |  | 1 (11.1%) |  | 1 (3.3%) |
| Missing (N) |  |  |  | 1 | 1 |
| ^a^Percentages calculated out of total patients in monotherapy or total patients in combination therapy  5-FU: 5-fluorouracil; CARB: carboplatin; CAPE: capecitabine; CISP: cisplatin; DOCE: docetaxel; EPI: epirubicin; IRIN: irinotecan; NIVO: nivolumab; OXAL: oxaliplatin; PAC: paclitaxel; RAMU: ramucirumab; TRAST: trastuzumab | | | | | |

**Table s3 Overall survival multivariate analysis results (excluding patients with ECOG PS missing)**

| **Analysis of Maximum Likelihood Estimates** | | | | | | | | |
| --- | --- | --- | --- | --- | --- | --- | --- | --- |
| **Parameter** |  | **Parameter**  **Estimate** | **Standard**  **Error** | **Chi-Square** | **Pr > ChiSq** | **Hazard**  **Ratio** | **95% Hazard Ratio Confidence**  **Limits** | |
| ECOG (ref=0 + 1. Fully active + Restricted) | 2 + 3. Ambulatory and capable | 0.40604 | 0.23958 | 28.722 | 0.0901 | 1.501 | 0.938 | 2.400 |
| N metastatic locations (ref=1 metastatic location) | More than one metastatic location | 0.45802 | 0.19642 | 54.378 | 0.0197 | 1.581 | 1.076 | 2.323 |
| Treatment regimen (ref= Mono-therapy) | Combination  Therapy | -0.30318 | 0.19197 | 24.942 | 0.1143 | 0.738 | 0.507 | 1.076 |

DF = 1 for each parameter

**Table s4 Progression-free survival multivariate analysis results (excluding patients with ECOG PS missing)**

| **Analysis of Maximum Likelihood Estimates** | | | | | | | | |
| --- | --- | --- | --- | --- | --- | --- | --- | --- |
| **Parameter** |  | **Para-meter**  **Estimate** | **Std**  **Error** | **Chi-Square** | **Pr > ChiSq** | **Hazard**  **Ratio** | **95% Hazard Ratio Confidence**  **Limits** | |
| Gender (ref=Male) | Female | 0.33014 | 0.19214 | 29.523 | 0.0858 | 1.391 | 0.955 | 2.027 |
| Nf metastatic locations (ref=1 metastatic location) | More than one metastatic location | 0.41117 | 0.18602 | 48.855 | 0.0271 | 1.509 | 1.048 | 2.172 |
| Treatment regimen (ref= Monotherapy) | Combination Therapy | -0.23595 | 0.18194 | 16.819 | 0.1947 | 0.790 | 0.553 | 1.128 |

DF = 1 for each parameter
